# Editorial: Electrical management of heart failure: shaping the future of cardiac pacing and electrophysiology

**DOI:** 10.3389/fcvm.2023.1325989

**Published:** 2023-11-02

**Authors:** Edoardo Bressi, Justin G. Luermans, Ahran D. Arnold, Domenico Grieco

**Affiliations:** ^1^ Department of Cardiovascular Sciences, Policlinico Casilino of Rome, Rome, Italy; ^2^ Department of Cardiology, Cardiovascular Research Institute Maastricht (CARIM), Maastricht University Medical Center, Maastricht, Netherlands; ^3^ National Heart and Lung Institute, Imperial College London, London, United Kingdom

**Keywords:** heart failure, conduction system pacing, atrial fibrillation ablation, ventricular tachiarrhythmias, remote monitoring

Editorial on the Research Topic Electrical management of heart failure: shaping the future of cardiac pacing and electrophysiology

In the relentless pursuit of mitigating the global burden of heart failure (HF), the cardiovascular community is perpetually exploring avant-garde strategies that promise improved life quality and enhanced survival rates for the affected population.

Over the years, pharmacological interventions have been the cornerstone of managing heart failure. However, as our understanding of the complex interplay between the electrical and mechanical aspects of cardiac function deepens, innovative cardiac pacing and electrophysiology approaches offer additional promising avenues to reshape the trajectory of heart failure therapy and improve patient outcomes (1).

This editorial on the electrical management of HF provides an incisive look into the current paradigm and contemplates future trajectories in these burgeoning fields.

Cardiac pacing technology has made substantial strides since its inception. Whether it began as a rescue measure to address bradyarrhythmias in the past decades, it has evolved into a potent therapeutic tool for HF (2).

The pivotal moment in the convergence of cardiac pacing and HF was the advent of biventricular cardiac resynchronization therapy (CRT) (3). CRT mitigates the dyssynchrony often observed in heart failure patients, especially those with left bundle branch block, by ensuring the synchronized contraction of the ventricles. Continually refined patient selection criteria, optimization of lead placement with multi-site pacing, and further technological enhancements are objects of continued research to amplify the benefits derived from conventional CRT (4).

In this scenario, the advent of the conduction system pacing (CSP), namely His-bundle pacing (HBP) and Left bundle branch area pacing (LBBAP), by preserving the heart's natural electrical pathways, offers more physiological ventricular activation and has revolutionized the pacing world (3, 5). Indeed, CSP showed promising preliminary evidence in reducing the risk of heart failure with adverse remodeling seen with long-term conventional right ventricular pacing (i.e., pacing-induced cardiomyopathy) and a valid alternative to conventional biventricular pacing (BVP) in those candidates for CRT (6, 7).

As the autonomic tone is impaired and dysregulated in heart failure patients, Cardiac Contractility Modulation (CCM) therapy emerged as another tool in the armamentarium, particularly in patients who remain symptomatic despite optimal medical therapy. This non-excitatory electrical therapy delivers signals to the heart during the absolute refractory period, enhancing myocardial contractility without increasing heart rate (8).

Finally, as technological advancements foster novel pacing pathways, adopting leadless pacemakers could offer broader therapeutic applications in HF management, complementing existing strategies to optimize cardiac function and enhance patient quality of life without the traditional lead-wire system (9).

At the same time, modern advancements in mapping systems and ablation catheters, particularly in understanding the electrical derangements in HF, have ushered in a new era in cardiac electrophysiology (10).

The milieu of interventions, primarily to manage atrial and ventricular arrhythmias in HF patients, has been subject to intensive research and evolution. Atrial fibrillation (AF) is the most common arrhythmia in HF, primarily causing or exacerbating an already compromised cardiac function, underscoring the imperative need for optimal management (10, 11). Novel ablation strategies, underpinned by a deeper understanding of the underlying electrophysiological substrates, triggers, and drivers, can offer a potential avenue to quell the perturbations instigated by AF, thereby alleviating the multifaceted challenges of managing HF (11).

Additionally, ventricular tachycardia (VT), another nemesis in the HF population, has witnessed the boon of advanced ablation techniques and substrate-based strategies, minimizing the reliance on antiarrhythmic drugs (12). Indeed, ventricular tachycardia and frequent ventricular ectopies can now be tackled using catheter ablation, sparing patients from defibrillator shocks due to arrhythmic storms or the potential occurrence of tachycardiomiopathy induced by frequent premature ventricular contractions (12–14).

Not lastly, cardioneuroablation is a relatively new catheter-based therapeutic procedure aimed at modulating the autonomic tone of the heart by ablating specific ganglionic plexi and nerve fibers. Adjusting the autonomic balance could enhance cardiac function, reduce arrhythmic episodes, and improve overall cardiac remodeling in HF patients (15).

The horizon beholds an interconnected era where digital health integrates seamlessly with cardiac pacing and electrophysiology. Remote monitoring and managing HF patients via implantable devices present a paradigm where real-time data is utilized to optimize therapeutic interventions, potentially reducing hospitalizations and enhancing patient outcomes (15). Furthermore, integrating artificial intelligence (AI) in managing, analyzing, and interpreting the vast arrays of data from these devices signifies a future where personalized and predictive medicine could become embedded within the standard of care (16).

Despite the significant promise, the road ahead is punctuated with challenges requiring meticulous attention. Ethical, regulatory, and technical aspects related to digital health and AI, patient and provider education, health disparities, and ensuring equitable access to advanced technologies are paramount considerations that must be addressed.

Investment in comprehensive research that spans bench to bedside, encompassing basic science to understand the underpinnings of electrical disturbances in HF, translational research to develop innovative interventions, and robust clinical trials to ascertain efficacy and safety is imperative. Furthermore, fostering collaborative initiatives that amalgamate expertise from cardiology, electrophysiology, engineering, data science, and other relevant disciplines would catalyze innovations and expedite their translation to clinical practice.

In reflection, the electric management of HF via advancements in cardiac pacing and electrophysiology embodies a luminous beacon of hope for individuals suffering from HF. By intertwining technological advancements with an intimate understanding of cardiac electrophysiology, a future where the morbidity and mortality of HF are significantly curtailed is not merely a utopian dream but a tangible reality.

The electrical management of HF represents, therefore, an exhilarating frontier in cardiovascular medicine. As we navigate this exciting era, this research collection aims to bring evidence on the foremost available novelties for handling patients with arrhythmic conditions related to HF and to shape a step forward in the therapeutical approach to heart failure.

## References

[1] Prinzen FW Auricchio A Mullens W Linde C Huizar JF. Electrical management of heart failure: from pathophysiology to treatment. Eur Heart J. (2022) 43(20):1917–27. 10.1093/eurheartj/ehac088 35265992 PMC9123241

[2] Chung MK Patton KK Lau CP Dal Forno ARJ Al-Khatib SM Arora V 2023 HRS/APHRS/LAHRS guideline on cardiac physiologic pacing for the avoidance and mitigation of heart failure. Heart Rhythm. (2023) 20(9):e17–91. 10.1016/j.hrthm.2023.03.1538 37283271 PMC11062890

[3] Ellenbogen KA Auricchio A Burri H Gold MR Leclercq C Leyva F The evolving state of cardiac resynchronization therapy and conduction system pacing: 25 years of research at EP europace journal. Europace. (2023) 25(8):euad168. 10.1093/europace/euad168 37622580 PMC10450796

[4] Nguyên UC Prinzen FW Vernooy K. Left ventricular lead placement in cardiac resynchronization therapy: current data and potential explanations for lack of benefit. Heart Rhythm. (2023):S1547-5271(23)02779-0. 10.1016/j.hrthm.2023.10.003 37806647

[5] Bressi E Grieco D Čurila K Zanon F Marcantoni L Cabrera JA Pacing of the specialized his-purkinje conduction system: “back to the future”. Eur Heart J Suppl. (2023) 25(Suppl C):C234–41. 10.1093/eurheartjsupp/suad047 37125312 PMC10132574

[6] Simader F Arnold A Whinnett Z. Comparison of methods for delivering cardiac resynchronization therapy: electrical treatment targets and mechanisms of action. Expert Rev Med Devices. (2023) 20(5):337–48. 10.1080/17434440.2023.2199925 37071055

[7] Bressi E Grieco D Luermans J Burri H Vernooy K. Conduction system pacing for cardiac resynchronization therapy: state of the art, current controversies, and future perspectives. Front Physiol. (2023) 14:1124195. 10.3389/fphys.2023.1124195 36711020 PMC9880410

[8] Masarone D Kittleson MM D'Onofrio A Falco L Fumarolo I Massetti M Basic science of cardiac contractility modulation therapy: molecular and electrophysiological mechanisms. Heart Rhythm. (2023):S1547-5271(23)02767-4. 10.1016/j.hrthm.2023.09.021 37769793

[9] Wijesuriya N De Vere F Mehta V Niederer S Rinaldi CA Behar JM. Leadless pacing: therapy, challenges and novelties. Arrhythm Electrophysiol Rev. (2023) 12:e09. 10.15420/aer.2022.41 37427300 PMC10326662

[10] Svennberg E Caiani EG Bruining N Desteghe L Han JK Narayan SM The digital journey: 25 years of digital development in electrophysiology from an europace perspective. EP Europace. (2023) 25(8):euad176. 10.1093/europace/euad176 PMC10450797 37622574

[11] Boersma L Andrade JG Betts T Duytschaever M Pürerfellner H Santoro F Progress in atrial fibrillation ablation during 25 years of europace journal. EP Europace. (2023) 25(9):euad244. 10.1093/europace/euad244 PMC10451004 37622592

[12] Natale A Zeppenfeld K Della Bella P Liu X Sabbag A Santangeli P Twenty-five years of catheter ablation of ventricular tachycardia: a look back and a look forward. EP Europace. (2023) 25(9):euad225. 10.1093/europace/euad225 37622589 PMC10451002

[13] Marcus GM. Evaluation and management of premature ventricular complexes. Circulation. (2020) 141(17):1404–18. 10.1161/CIRCULATIONAHA.119.042434 32339046

[14] Huizar JF Kaszala K Tan A Koneru J Mankad P Kron J Abnormal conduction-induced cardiomyopathy: JACC review topic of the week. J Am Coll Cardiol. (2023) 81(12):1192–200. 10.1016/j.jacc.2023.01.040 36948737 PMC10715964

[15] Pachon JC Pachon EI Aksu T Gopinathannair R Kautzner J Yao Y Cardioneuroablation: where are we at? Heart Rhythm O2. (2023) 4(6):401–13. 10.1016/j.hroo.2023.02.007 37361615 PMC10287999

[16] Mastoris I Gupta K Sauer AJ. The war against heart failure hospitalizations: remote monitoring and the case for expanding criteria. Cardiol Clin. (2023) 41(4):557–73. 10.1016/j.ccl.2023.06.001 37743078

